# A combined method of optimized learning vector quantization and neuro-fuzzy techniques for predicting unified Parkinson's disease rating scale using vocal features

**DOI:** 10.1016/j.mex.2024.102553

**Published:** 2024-01-05

**Authors:** Waleed Abdu Zogaan, Mehrbakhsh Nilashi, Hossein Ahmadi, Rabab Ali Abumalloh, Mesfer Alrizq, Hamad Abosaq, Abdullah Alghamdi

**Affiliations:** a Department of Computer Science, Faculty of Computer Science and Information Technology, Jazan University, Jazan 45142, Saudi Arabia; b UCSI Graduate Business School, UCSI University, Cheras, Kuala Lumpur 56000, Malaysia; c Centre for Global Sustainability Studies (CGSS), Universiti Sains Malaysia, Penang, 11800, Malaysia; d Centre for Health Technology, Faculty of Health, University of Plymouth, Plymouth, PL4 8AA, UK; e Faculty of Health, University of Plymouth, Plymouth, PL4 8AA, UK; f Department of Computer Science and Engineering, Qatar University, Doha 2713, Qatar; g Information Systems Department, College of Computer Science and Information Systems, Najran University, Najran, Saudi Arabia; h Scientific and Engineering Research Center (SERC), Najran University, Najran, Saudi Arabia; i Computer Science Department, College of Computer Science and Information Systems, Najran University, Najran, Saudi Arabia

**Keywords:** Parkinson's disease, Neuro-fuzzy, Optimized learning rate, Motor-UPDRS, Total-UPDRS, Learning vector quantization, A Combined Method of Optimized Learning Vector Quantization and Neuro-Fuzzy Techniques

## Abstract

Parkinson's Disease (PD) is a common disorder of the central nervous system. The Unified Parkinson's Disease Rating Scale or UPDRS is commonly used to track PD symptom progression because it displays the presence and severity of symptoms. To model the relationship between speech signal properties and UPDRS scores, this study develops a new method using Neuro-Fuzzy (ANFIS) and Optimized Learning Rate Learning Vector Quantization (OLVQ1). ANFIS is developed for different Membership Functions (MFs). The method is evaluated using Parkinson's telemonitoring dataset which includes a total of 5875 voice recordings from 42 individuals in the early stages of PD which comprises 28 men and 14 women. The dataset is comprised of 16 vocal features and Motor-UPDRS, and Total-UPDRS. The method is compared with other learning techniques. The results show that OLVQ1 combined with the ANFIS has provided the best results in predicting Motor-UPDRS and Total-UPDRS. The lowest Root Mean Square Error (RMSE) values (UPDRS (Total)=0.5732; UPDRS (Motor)=0.5645) and highest R-squared values (UPDRS (Total)=0.9876; UPDRS (Motor)=0.9911) are obtained by this method. The results are discussed and directions for future studies are presented.

i. ANFIS and OLVQ1 are combined to predict UPDRS.
ii. OLVQ1 is used for PD data segmentation.
iii. ANFIS is developed for different MFs to predict Motor-UPDRS and Total-UPDRS.

## Graphical abstract

**Figure:**
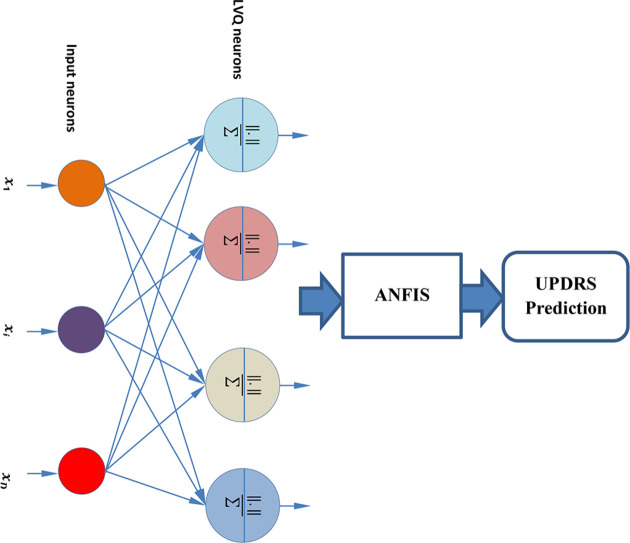

Specifications table

**Table:** 

Subject area:	Neuroscience
More specific subject area:	Parkinson's disease
Name of your method:	A Combined Method of Optimized Learning Vector Quantization and Neuro-Fuzzy Techniques
Name and reference of original method:	Tsanas, A., Little, M., McSharry, P., & Ramig, L. (2009). Accurate telemonitoring of Parkinson's disease progression by non-invasive speech tests. Nature Precedings, 1-1.
Resource availability:	https://archive.ics.uci.edu/dataset/189/parkinsons+telemonitoring

## Method details

Tracking the progression of Parkinson's Disease (PD) remotely permits patients to be monitored without their physical presence in the clinic. Patients typically collect data at home using monitoring devices, which are then transmitted to the clinic *via* telephone or internet connections. The use of remote tracking techniques offers a promising solution for the management of a growing patient population, especially in situations where geographical constraints or limited resources make traditional clinic-based care challenging. The UPDRS is commonly used to track PD symptom progression because it displays the presence and severity of symptoms. It has been suggested to track the progression of PD symptoms by linking measures of PD dysphonia to the Motor-UPDRS and Total-UPDRS [1,2]. Machine learning algorithms have the potential to assist physicians in both diagnosing Parkinson's disease and quantifying its progression by extracting valuable patterns from processed data [3]. To model the relationship between speech signal properties and UPDRS scores, various machine learning techniques have been employed such as Support Vector Machines (SVMs) [4], [5], [6], Adaptive Neuro-Fuzzy Inference System [7,8], Support Vector Regression (SVR) [9], Neural Networks [10], [11], [12], [13], [14], [15], [16], [17], [18], and Gaussian Process Regression [19].

In contrast with the previous method for PD diagnosis which relies solely on supervised learning techniques, this study develops a new method using Adaptive Neuro-Fuzzy Inference System (ANFIS) and Optimized Learning Rate Learning Vector Quantization (OLVQ1). ANFIS models are developed for different Membership Functions (MFs) with a hybrid learning algorithm. The method is evaluated using Parkinson's telemonitoring dataset which includes a total of 5875 voice recordings from 42 individuals in the early stages of PD which comprises 28 men and 14 women. The dataset is comprised of 16 vocal features and Motor-UPDRS, and Total-UPDRS. The method is compared with the Support Vector Regression (SVR), ANFIS, Gaussian Process Regression (GPR) and the combination of OLVQ1 with ANFIS for different Triangular MF, Trapezoidal MF, Generalized Bell MF, and Gaussian MF.

To model the relationship between speech signal properties and UPDRS scores, this study develops a new method using ANFIS and OLVQ1. ANFIS is developed for different MFs. These techniques are introduced in the following sections.

### LVQ

**Fig. 1:**
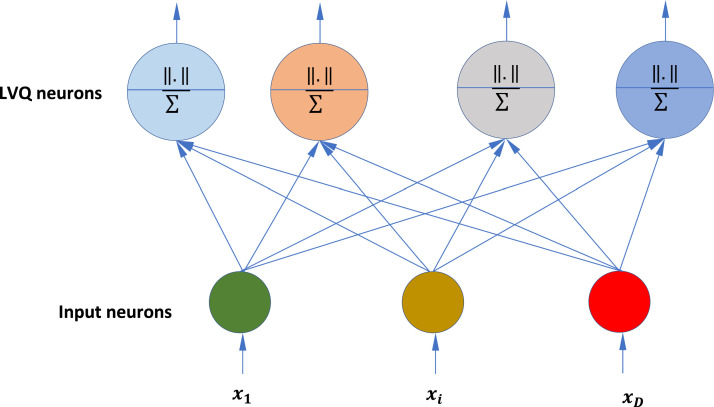
LVQ network.

LVQ is an algorithm for supervised competitive neural network learning [20]. The LVQ network is illustrated in Fig. 1. The network includes two layers: an input layer and a hidden layer with J neurons. The input layer receives input examples and the hidden layer is considered as code vectors or prototypes. These prototype vectors, indicated by $c_{1,...,}c_{J}$, partition the input space into *J* distinct regions known as Voronoi cells. During the training phase, a training set, which is denoted by $L=\{x_{\mu },y\}\;:\;\mu =1,2,\ldots ,M$, is iteratively given to the LVQ network. $x_{\mu }$ are the presented training instances.The LVQ algorithm initializes $c_{j}$, j=1,…,J, by random selection of J instances from the dataset in training set L. In each iteration of the network training, the position of a prototype $c_{j}$ is adjusted based on its distances to$\;x_{\mu }$. If a prototype $c_{j}\;$and the input sample $x_{\mu }$ belong to the same class, the prototype moves towards $x_{\mu }$. On the other hand, if they belong to different classes, the prototype moves in the opposite direction. This process of updating the prototype locations continues iteratively.

In the classification stage, an instance x is given the label of the class corresponding to its nearest prototype$\;c_{j*}$, where the nearest prototype can be defined in Eq. (1) as:

(1) $$j^{*}=arg\;\text{mi}n_{1\leq j\leq J}∥x-c_{j}∥.$$

LVQ1 is the first developed LVQ network. In LVQ1, during each iteration t and for each example $x_{\mu }$, the first step involves calculating the distance between training instances $x_{\mu }$ and all prototypes $c_{j}$.

(2) $$d_{j}=∥x-c_{j}∥$$

Accordingly, we can define the index of the winning prototype $\;c_{j^{*}}\;$as:

(3) $$j^{*}=arg\;\text{mi}n_{1\leq j\leq J}\;d_{j}.$$

Then, we have:

(4) $$c_{j}\left(t+1\right)=\left\{ \begin{array}{c} c_{j}\left(t\right)+\eta \left(t\right)\left(x_{\mu }-c_{j}\left(t\right)\right)\;if\;class\;\left(c_{j}\right)=class\left(x_{\mu }\right),\;j=j^{*}\\ c_{j}\left(t\right)-\eta \left(t\right)\left(x_{\mu }-c_{j}\left(t\right)\right)\;if\;class\;\left(c_{j}\right)=class\left(x_{\mu }\right),\;j\neq j^{*}\\ c_{j}\left(t\right),\;j\neq j^{*} \end{array}\right.$$

If $\;c_{j^{*}}$ and $x_{\mu }$ share the same class, the winning neuron is adjusted towards $x_{\mu }$. Conversely, if they belong to different classes, the winning neuron is pushed away. The adjustment of the winning neuron's position is influenced by the global learning rate η(t), which can either remain constant or decrease over time t, with values ranging from 0 to 1.

Optimized learning rate LVQ1 or OLVQ1 is an enhanced variation of LVQ1 that incorporates individual learning rates $\eta_{j}\left(t\right)\;$for each prototype $c_{j}\left(t\right)\;$in the learning rule, rather than utilizing a global learning rate η(t). OLVQ1 aims to expedite the convergence process. The local learning rate $\eta_{j}\left(t\right)\;$is defined as:

(5) $$\eta_{j}\left(t\right)=\;\text{min}\;\left(\frac{\eta_{j}\left(t-1\right)}{s\left(t\right)\eta_{j}\left(t-1\right)+1},\;\eta_{\;\text{max}\;}\right)$$

The initial learning rate, denoted as $\eta_{j}\left(0\right)$, is used as the starting point for each prototype's learning rate. The value of s(t)is determined based on the class membership of $c_{j}\;$and x, with s(t)equal to 1 if they belong to the same class, and s(t) equal to -1 otherwise. It is important to note that the learning rate $\eta_{j}\left(t\right)$ has the potential to increase. To prevent uncontrolled growth, an upper bound $\eta_{\;\text{max}}$, which falls within the range of 0 to 1, is defined for each $\eta_{j}\left(t\right)$.

### ANFIS

**Fig. 2:**
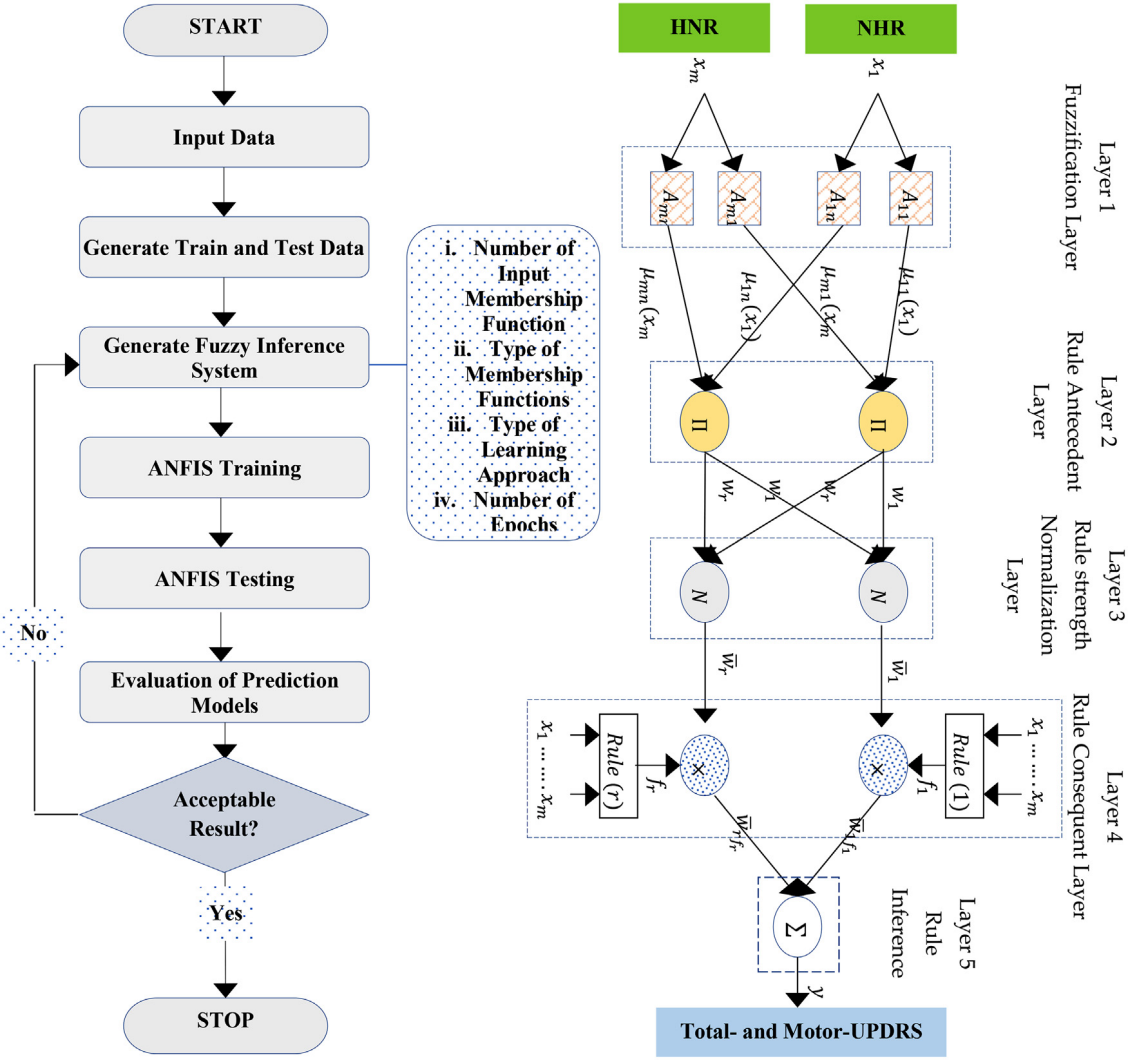
Flowchart of five-layer ANFIS model.

In this study, the Adaptive Neuro-Fuzzy Inference System (ANFIS) [21] is employed to predict the Total- and Motor-UPDRS using a set of speech signals (dysphonia measures). ANFIS combines fuzzy logic and neural network methodologies and is commonly utilized in prediction tasks, particularly in the domain of tourism and hospitality. By establishing mappings between input and output variables, ANFIS generates optimal membership functions that enable accurate predictions based on a set of fuzzy rules. ANFIS offers various types of Membership Functions (MF), including Triangular MF, Trapezoidal MF, Generalized Bell MF, and Gaussian MF. This research employs all of these MFs in ANFIS modeling to predict the UPDRS score. ANFIS is structured into five distinct layers which is illustrated in Fig. 2.

## Data analysis and results

The Parkinson's telemonitoring dataset was developed through a collaboration between Athanasios Tsanas and Max Little from the University of Oxford, along with 10 medical centers in the US and Intel Corporation. It was designed to work in conjunction with the AHTD telemonitoring device, specifically created for recording speech signals from individuals with Parkinson's disease (PD) [1]. This dataset became available on the UCI Machine Learning Archive in October 2009. The dataset includes recordings from 42 individuals in the early stages of PD which comprises 28 men and 14 women. There were a total of 5875 voice recordings because each patient contributed approximately 200 voice recordings, making the total number of voice recordings 5875. The recordings were made with the patients maintaining the vowel sound /a/ while the recordings were being made. The dataset is comprised of 26 attributes, which include a variety of information such as the subject's number, age, gender, time interval from baseline recruitment data, motor-UPDRS, total-UPDRS, and 16 biomedical voice measures, also known as vocal features. In addition, the Parkinson's telemonitoring dataset includes 16 vocal features (see Table 1). The vocal characteristics include a wide variety of measurements such as jitter, shimmer, HNR, and NHR.

**Table 1:** Parkinson's telemonitoring dataset for method evaluation.

Variable	Feature	Min	Max	Mean	SD
F1	MDVP:Jitter (%)	8E-4	0.1	0.006	0.006
F2	MDVP:Jitter (Abs)	2E-6	4E-4	4E-5	3E-5
F3	MDVP:Jitter:RAP	3E-4	0.057	0.003	0.003
F4	MDVP:Jitter:PPQ5	4E-4	0.069	0.003	0.004
F5	Jitter:DDP	10E-4	0.173	0.009	0.009
F6	MDVP:Shimmer	0.003	0.269	0.034	0.026
F7	MDVP:Shimmer (dB)	0.026	2.107	0.311	0.230
F8	Shimmer:APQ3	0.002	0.163	0.017	0.013
F9	Shimmer:APQ5	0.002	0.167	0.020	0.017
F10	Shimmer:APQ11	0.003	0.276	0.028	0.020
F11	Shimmer:DDA	0.005	0.488	0.052	0.040
F12	NHR	3E-4	0.749	0.032	0.060
F13	HNR	1.659	37.875	21.679	4.291
F14	RPDE	0.151	0.966	0.541	0.101
F15	DFA	0.514	0.866	0.653	0.071
F16	PPE	0.022	0.732	0.220	0.092
-	Motor-UPDRS (baseline)	6	36	19.42	8.12
-	Motor-UPDRS (after three months)	6	38	21.69	9.18
-	Motor-UPDRS (after six months)	5	41	29.57	9.17
-	Total-UPDRS (baseline)	8	54	26.39	10.8
-	Total-UPDRS (after three months)	7	55	29.36	11.82
-	Total-UPDRS (after six months)	7	54	29.57	11.92

The scores on the Motor-UPDRS and the Total-UPDRS (as two outputs of the dataset) have been evaluated at the beginning of the trial, after three months, and after six months of treatment. Voice recordings, on the other hand, were collected on a weekly basis. The Motor-UPDRS scores and the total-UPDRS scores were linearly interpolated so that we could ensure that our data were consistent. The baseline, three-month, and six-month UPDRS scores are presented in Table 1 of the original research publication. Additionally, corresponding feature labels and concise explanations for each measurement are included in this table. Furthermore, some fundamental statistics regarding the dataset are provided in Table 1. This dataset has been widely used by researchers [1,[22], [23], [24]] in the field of Parkinson's disease to develop algorithms for the early detection and monitoring of PD symptoms based on vocal characteristics.

**Fig. 3:**
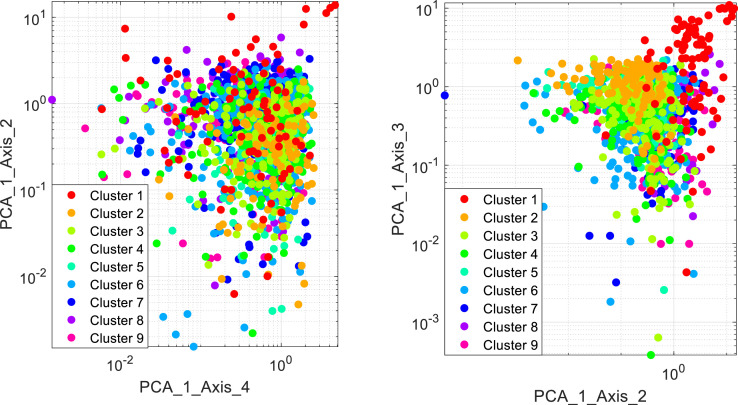
The visualization of clusters.

The data were clustered using LVQ. The results of data clustering are shown in Table 2. Nine clusters were generated from the Parkinson's telemonitoring dataset. The clusters are visualized in Fig. 3 using different principal components generated by principal components analysis.

**Table 2:** Cluster centroids.

Attribute	LVQ-Cluster 1 (556)	LVQ-C2 (432)	LVQ- Cluster 3 (583)	LVQ- Cluster 4 (633)	LVQ- Cluster 5 (264)	LVQ- Cluster 6 (987)	LVQ- Cluster 7 (995)	LVQ- Cluster 8 (601)	LVQ- Cluster 9 (824)
F1	0.015079	0.002467	0.003661	0.004125	0.003126	0.004865	0.006724	0.008359	0.005603
F2	0.000101	0.000013	0.000024	0.000028	0.000019	0.000034	0.000053	0.000066	0.000041
F3	0.007791	0.001107	0.001668	0.001924	0.001424	0.002274	0.003234	0.004177	0.002671
F4	0.009122	0.001199	0.001802	0.002079	0.001565	0.002445	0.003486	0.004368	0.002882
F5	0.023372	0.003320	0.005005	0.005774	0.004272	0.006821	0.009702	0.012532	0.008012
F6	0.085802	0.013397	0.019529	0.022005	0.016389	0.026308	0.037145	0.046450	0.031529
F7	0.773610	0.124829	0.181396	0.204896	0.151977	0.241459	0.339030	0.423592	0.287655
F8	0.042726	0.006358	0.009511	0.010851	0.007907	0.013172	0.019048	0.024264	0.016079
F9	0.052953	0.007381	0.011043	0.012665	0.009304	0.015284	0.021987	0.027507	0.018582
F10	0.066007	0.010789	0.016089	0.018300	0.013637	0.021838	0.030289	0.037422	0.025904
F11	0.128179	0.019074	0.028532	0.032554	0.023722	0.039517	0.057145	0.072793	0.048236
F12	0.142446	0.007027	0.012584	0.014355	0.009695	0.018030	0.027624	0.039345	0.022522
F13	13.049545	29.022574	25.433160	24.203379	26.684591	22.778716	19.727566	17.828852	21.303484
F14	0.666227	0.421046	0.479681	0.495495	0.446748	0.525615	0.581591	0.611050	0.549623
F15	0.677777	0.598035	0.628642	0.636257	0.611311	0.647959	0.677550	0.689234	0.660225
F16	0.358831	0.112652	0.154981	0.173169	0.135406	0.197310	0.253635	0.294498	0.220981

### Method evaluation

The experiment was conducted using Microsoft Windows 10 Pro on a laptop equipped with an Intel(R) Core(TM) i7-6700HQ CPU running at 2.60 GHz, with four cores and eight logical processors. To prevent overfitting, a 10-fold cross-validation approach was employed during the training of the LVQ and ANFIS models. The method is evaluated using two metrics: RMSE and R^2^. The formulas for these metrics are presented in Eqs. (6) and (7).

(6) $$\mathrm{RMSE}=\sqrt{\frac{\sum_{i=1}^{N}{\left(\mathrm{Actual}\;O_{i}-{\hat{\mathrm{Predicted}\;O}}_{i}\right)}^{2}}{N}}$$

(7) $$R^{2}=1-\frac{\sum_{i=1}^{N}{\left(\mathrm{Actual}\;O_{i}-{\hat{\mathrm{Predicted}\;O}}_{i}\right)}^{2}}{\sum_{i=1}^{N}{\left(\mathrm{Actual}\;O_{i}-\bar{\mathrm{Actual}\;O_{i}}\right)}^{2}}$$

where N is the number of instances in the LVQ cluster, $\mathrm{Actual}\;O_{i}\;$denotes the Total- and Motor-UPDRS, ${\hat{\mathrm{Predicted}\;\mathrm{Sf}}}_{i}$ denotes the predicted Total- and Motor-UPDRS, $\bar{\mathrm{Actual}\;O_{i}}$ is the mean value ofActualO.

**Fig. 4:**
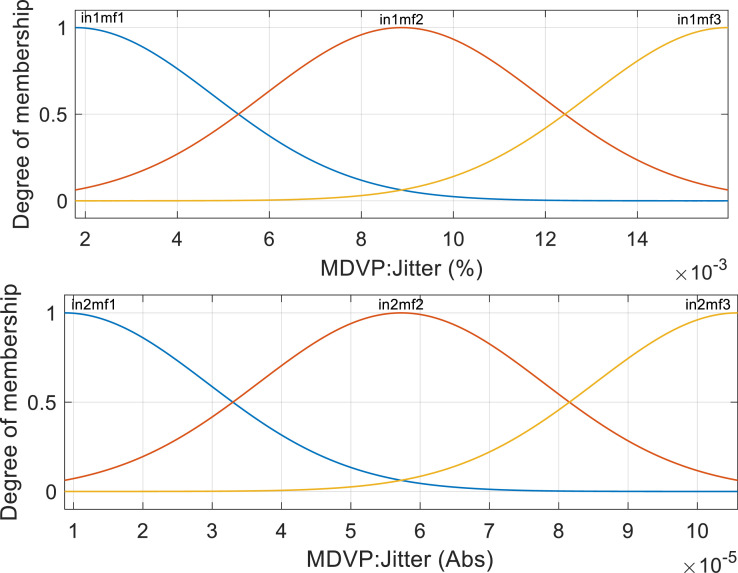
Gaussian MFs.

**Fig. 5:**
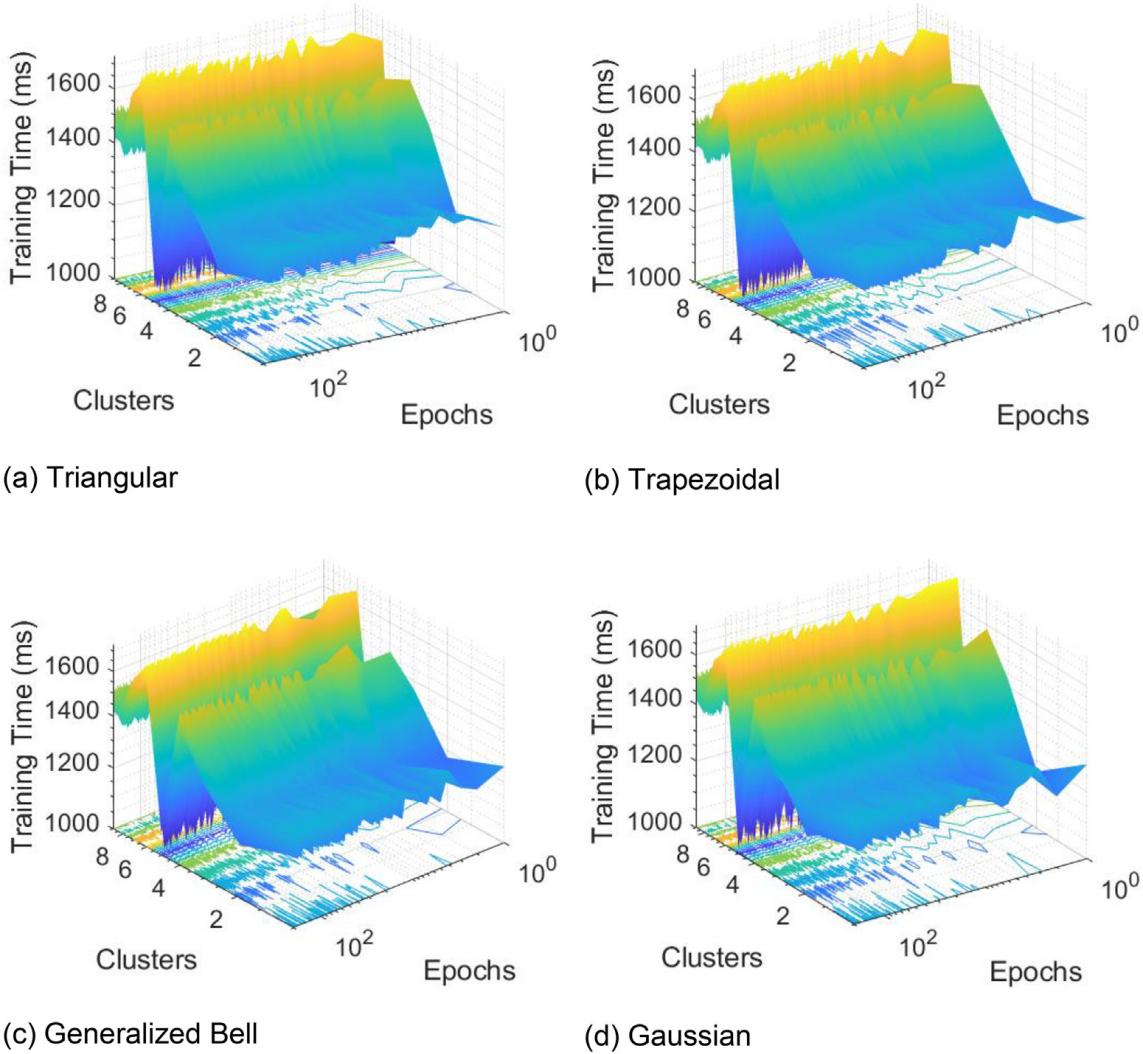
Training times in 200 epochs for different MFs in all clusters.

**Fig. 6:**
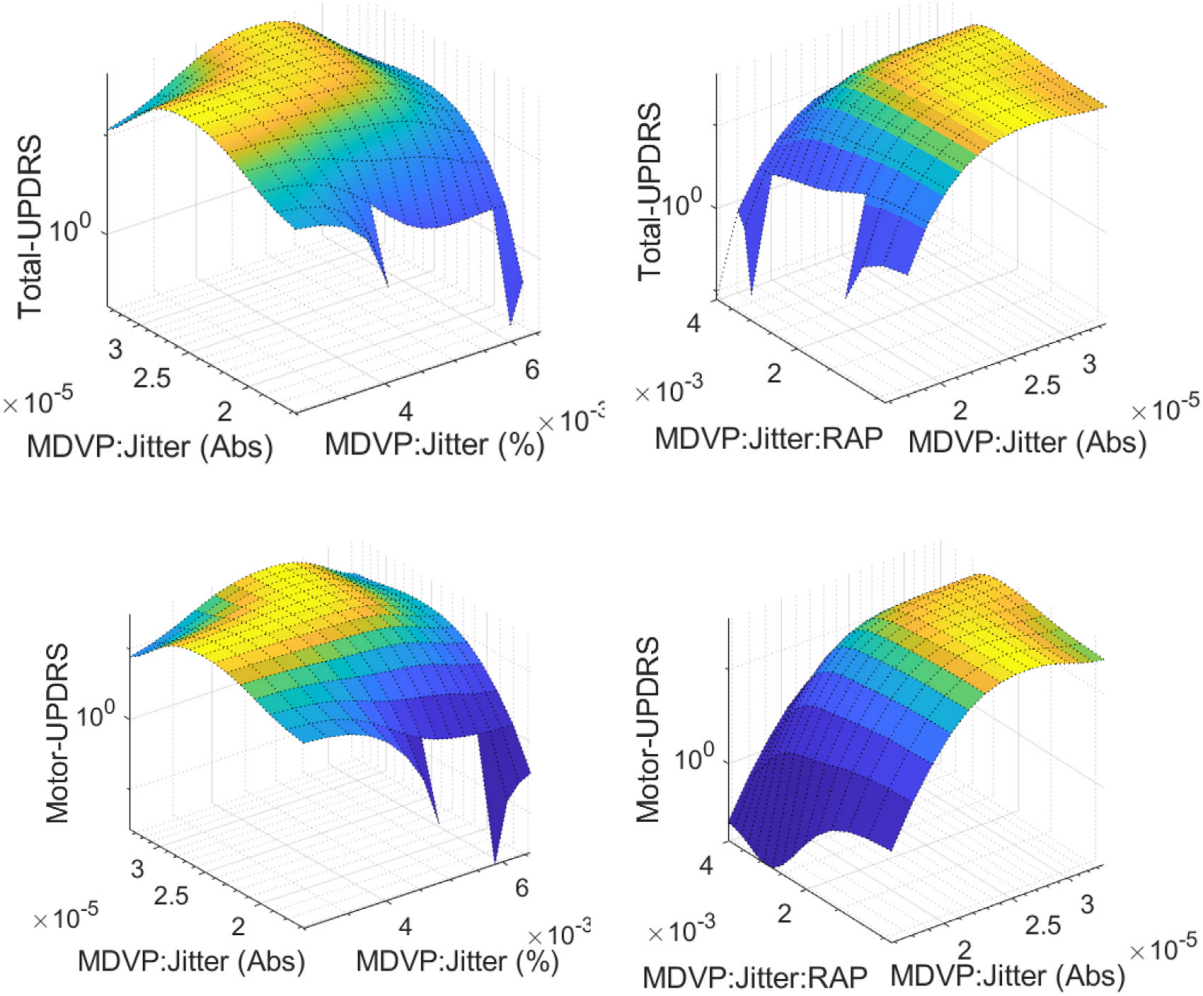
3D visualization of relationship between inputs and outputs in ANFIS.

The data was divided into 10 equal parts, where nine parts were used for training the model and the remaining part was used for testing. For example, the RMSE was calculated for each fold. This process was repeated for all ten folds. By averaging the RMSE values across all folds, an estimate of the model's overall performance was obtained. The nine models were evaluated based on their RMSE and correlation coefficients. A higher value of R^2^ indicates a better fit of the model. Conversely, lower values of RMSE indicate superior performance by the predictor. ANFIS was performed on the clusters to construct the prediction models. Different membership functions were used in ANFIS (i.e., Triangular MF, Trapezoidal MF, Generalized Bell MF, and Gaussian MF). An example of Gaussian MF is presented in Fig. 4. For each variable three membership functions were considered. The RMSE and R-squared values were obtained for each model and the average values were calculated for methods comparisons. In Fig. 5, we present the training times in 200 epochs for different MFs in all clusters. The 3D visualization of some relationships between inputs and outputs in ANFIS models are showing in Fig. 6.

The results of the method evaluation are presented in Table 3. We present the results for Motor-UPDRS and the Total-UPDRS for RMSE and R^2^. We perform the method evaluation for different methods, SVR, ANFIS, Gaussian Process Regression (GPR) and the combination of OLVQ1 with ANFIS for different Triangular MF, Trapezoidal MF, Generalized Bell MF, and Gaussian MF. The results show that OLVQ1 combined with the ANFIS has provided the best results in predicting Motor-UPDRS and Total-UPDRS. In addition, in relation to Trapezoidal MF, Generalized Bell MF, and Triangular MF, Gaussian MF provides the best results. The lowest RMSE values (UPDRS (Total)=0.5732; UPDRS (Motor)=0.5645) and highest R-squared values (UPDRS (Total)=0.9876; UPDRS (Motor)=0.9911) are obtained by this method. This evaluation was also performed for the LVQ1+ANFIS method which used Gaussian MF. The results are close to the results of OLVQ1+ANFIS with Triangular MF. Furthermore, when comparing the results of ANFIS and OLVQ1-ANFIS methods, there is a significant difference between the obtained accuracies, indicating that the use of OLVQ1 as a clustering technique is able to improve the efficiency of the ANFIS models in predicting Motor-UPDRS and the Total-UPDRS.

**Table 3:** Method evaluation.

Method	UPDRS	RMSE	R2
SVR	UPDRS (Total)	0.8781	0.8625
	UPDRS (Motor)	0.8656	0.8662
GPR	UPDRS (Total)	0.8621	0.8718
	UPDRS (Motor)	0.8568	0.8778
ANFIS	UPDRS (Total)	0.8469	0.8823
	UPDRS (Motor)	0.8396	0.8874
OLVQ1+ANFIS (Triangular MF)	UPDRS (Total)	0.6188	0.9522
	UPDRS (Motor)	0.6124	0.9546
OLVQ1+ANFIS (Trapezoidal MF)	UPDRS (Total)	0.6098	0.9621
	UPDRS (Motor)	0.6021	0.9689
OLVQ1+ANFIS (Generalized Bell MF)	UPDRS (Total)	0.5986	0.9723
	UPDRS (Motor)	0.5875	0.9764
OLVQ1+ANFIS (Gaussian MF)	UPDRS (Total)	0.5732	0.9876
	UPDRS (Motor)	0.5645	0.9911
LVQ1+ANFIS (Gaussian MF)	UPDRS (Total)	0.7133	0.9412
	UPDRS (Motor)	0.7028	0.9481

The outcome of our evaluation on the dataset also demonstrated that the method which used GPR has performed better predictions for the Total-UPDRS and Motor-UPDRS. Overall, it is concluded that the optimized learning rate LVQ1 has a significant advantage compared to the LVQ1 combined with ANFIS in predicting UPDRS for tracking PD progression. Note that, the RBF (Radial Basis Function) kernel was used in the SVR method. In addition, GPR used a squared exponential kernel for constructing the prediction models. ANFIS was trained for 200 epochs and with the use of a hybrid learning approach for all models.

## Conclusion

Parkinson's disease is a disorder that affects the central nervous system that, over time, reduces a person's mobility and negatively impacts their overall quality of life. The diagnosis of PD at an early stage is of the utmost significance since it permits rapid medical intervention. The method developed by machine learning plays a critical part in this process. They help the creation of diagnostic instruments that are non-invasive and cost-effective. For PD detection, machine learning algorithms are able to build reliable predictive models because they can analyze a wide variety of data kinds such as medical records, brain scans, and voice samples. These models provide assistance to medical professionals in spotting minor shifts in symptoms, which in turn makes it easier to initiate early intervention and develop individualized treatment programs. This research has aimed to develop a new method based on machine learning techniques for PD diagnosis. The method was developed using OLVQ1 and ANFIS machine learning techniques and evaluated using the Parkinson's telemonitoring dataset. Using LVQ, nine clusters were detected from the PD data. The ANFIS models were constructed on each cluster of LVQ to predict Motor-UPDRS and the Total-UPDRS. We performed several comparisons between this method and the LVQ1+ANFIS, SVR, ANFIS, and GPR, as well as the combination of OLVQ1 and ANFIS. According to the findings, the combination of the OLVQ1 and the ANFIS yielded the best results in predicting the Motor-UPDRS and the Total-UPDRS. In addition, the Gaussian MF obtained the best results with the smallest RMSE values (UPDRS (Total)=0.5732; UPDRS (Motor)=0.5645) and the highest R-squared values (UPDRS (Total)=0.9876; UPDRS (Motor)=0.9911) compared to the other MFs. This work includes several limitations which can be taken into account in developing new methods for PD diagnosis. First, this study has developed the method without the use of feature selection methods. They can be effective in investigating the relationship between vocal features and Motor-UPDRS and Total-UPDRS. In addition, feature selection can be an important phase of developing ANFIS models as when the number of features increases, there may be difficulty in the appropriate construction of prediction models by ANFIS. Second, ANFIS can be extended for incremental learning which can significantly increase the efficacy of the proposed method. Third, our method can be extended for ensemble learning. Ensemble learning techniques have demonstrated to be more stable in relation to individual learning techniques. Finally, this study found that the combination of clustering and prediction learning techniques can be effective in modeling predictive approaches for PD diagnosis, therefore, the clustering methods can be optimized and used in the proposed method for better data clustering.

## Ethics statements

Human subjects: Not Applicable.

Animal experiments: Not Applicable.

Data collected from social media platforms: Not Applicable.

## CRediT authorship contribution statement

**Waleed Abdu Zogaan:** Writing – original draft, Writing – review & editing, Visualization. **Mehrbakhsh Nilashi:** Conceptualization, Methodology, Software, Formal analysis, Investigation, Resources, Data curation, Writing – original draft, Writing – review & editing, Visualization. **Hossein Ahmadi:** Conceptualization, Investigation, Writing – review & editing, Visualization, Software. **Rabab Ali Abumalloh:** Writing – review & editing, Visualization, Software. **Mesfer Alrizq:** Writing – review & editing, Visualization, Software. **Hamad Abosaq:** Writing – review & editing, Visualization, Software. **Abdullah Alghamdi:** Writing – review & editing, Visualization, Software.

## Declaration of competing interest

The authors declare that they have no known competing financial interests or personal relationships that could have appeared to influence the work reported in this paper.
